# Supplementary material for: H2B ubiquitination recruits FACT to maintain a stable altered nucleosome state for transcriptional activation
Source: Nat Commun. 2023 Feb 10;14:741. doi: 10.1038/s41467-023-36467-3 (PMC9918737; doi:10.1038/s41467-023-36467-3)
Supplement: Supplementary file 1 — Supplementary informationn [file 41467_2023_36467_MOESM1_ESM.pdf]

Supplemental Information for

H2B ubiquitination recruits FACT to maintain a stable altered nucleosome state for transcriptional activation.

Anfeng Luo[1,6], Jingwei Kong[2,4,6], Jun Chen[1,6], Xue Xiao[2,6], Jie Lan[3], Xiaorong Li[3], Cuifang Liu[3], Peng-Ye Wang[2,4,5], Guohong Li[3,4], Wei Li[2,3,5*] and Ping Chen[1,3*]

[1] Department of Immunology, School of Basic Medical Sciences, Beijing Key Laboratory for Tumor Invasion and Metastasis, Capital Medical University, Beijing 100069, China;

[2] National Laboratory for Condensed Matter Physics and Key Laboratory of Soft Matter Physics, Institute of Physics, Chinese Academy of Sciences, Beijing 100101, China;

[3] National Laboratory of Biomacromolecules, CAS Center for Excellence in Biomacromolecules, Institute of Biophysics, Chinese Academy of Sciences, Beijing 100190, China;

[4] University of Chinese Academy of Sciences, Beijing 100049, China;

[5] Songshan Lake Materials Laboratory, Dongguan, Guangdong 523808, China

[6] These authors contributed equally to this work.

*Correspondence to: Wei Li: weili007@iphy.ac.cn; Ping Chen: chenping@ccmu.edu.cn;

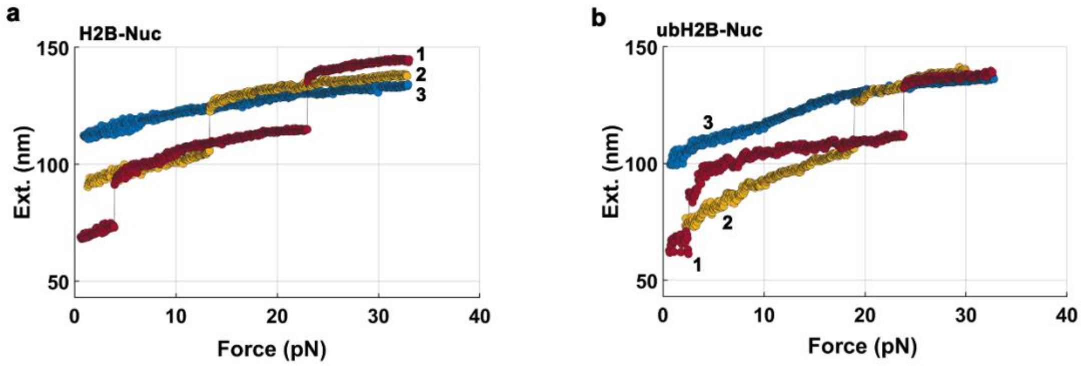

Supplementary Fig. 1: The effect of the ubH2B on the stability of nucleosome.

The 2D-plots of the repeated stretching measurements of H2B-nucleosome (a) and ubH2B-nucleosome (b). In each stretching cycle, the force is increased up to 32 pN. Source data are provided as a Source Data file.

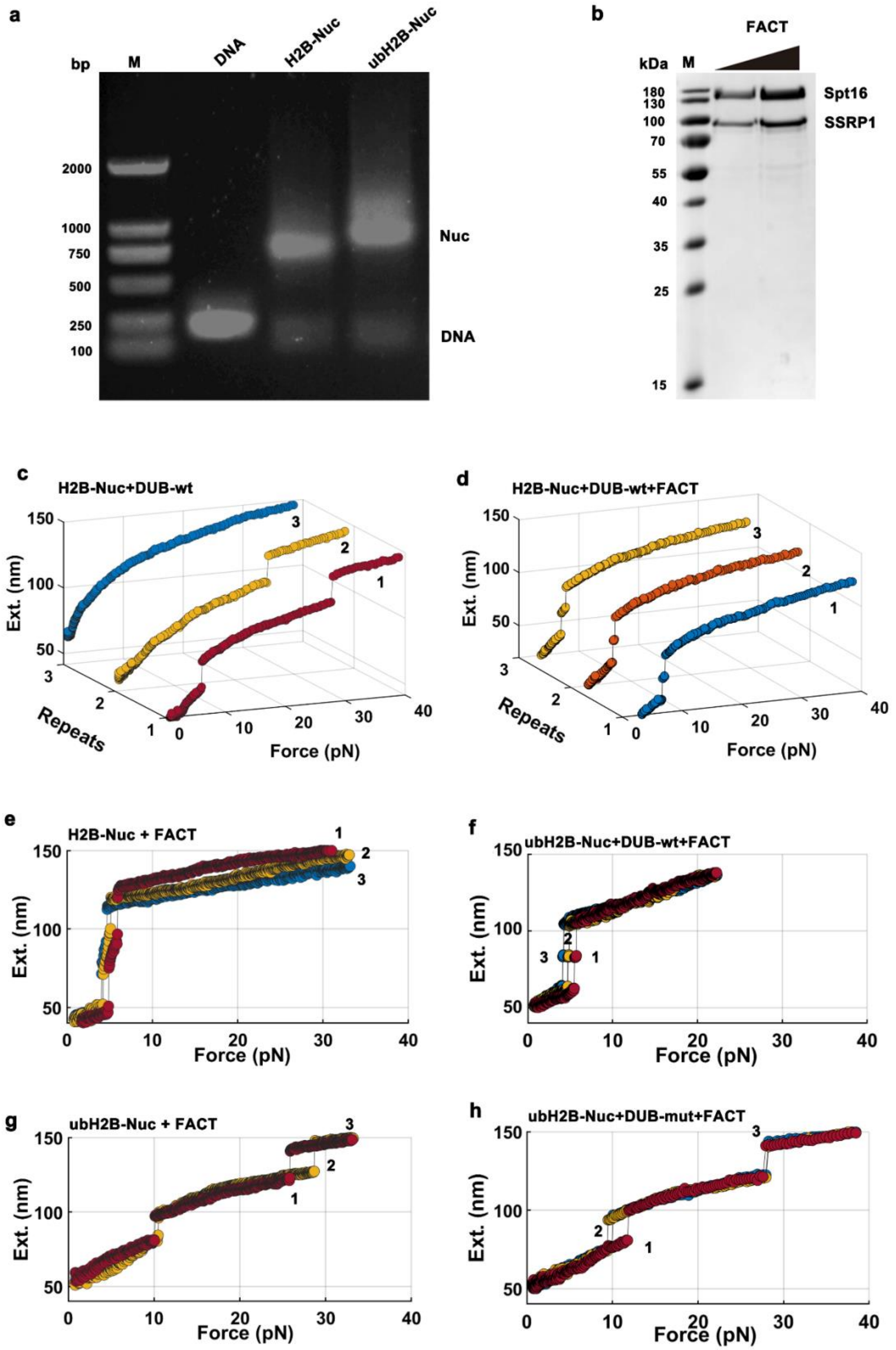

Supplementary Fig. 2: ubH2B recruits FACT to stabilize and maintain nucleosome structure.

(a) 1% agarose gel electrophoresis analysis of reconstituted H2B-nucleosome (H2B-Nuc) and ubH2B-nucleosome (ubH2B-Nuc) for the mono-nucleosome pull-down assay, with free DNA template (DNA) as control. The data are representative of *n*=3 biologically independent experiments. The first lane is the marker (M). (b) SDS-PAGE analysis of the purified FACT complex. The data are representative of *n*=3 biologically independent experiments. The first lane is the marker (M). (c-d) The typical repeated stretching measurements of H2B-nucleosome incubated with DUB-wt (c) and with both DUB-wt and FACT (d). The DUB has no effect on the nucleosome dynamics and the FACT's function on nucleosome. (e-h) The 2D-plots of the repeated stretching measurements of H2B-nucleosome with FACT (e), ubH2B-nucleosome with DUB-wt and FACT (f), ubH2B-nucleosome with FACT (g), and ubH2B-nucleosome with DUB-mut and FACT (h). Source data are provided as a Source Data file.

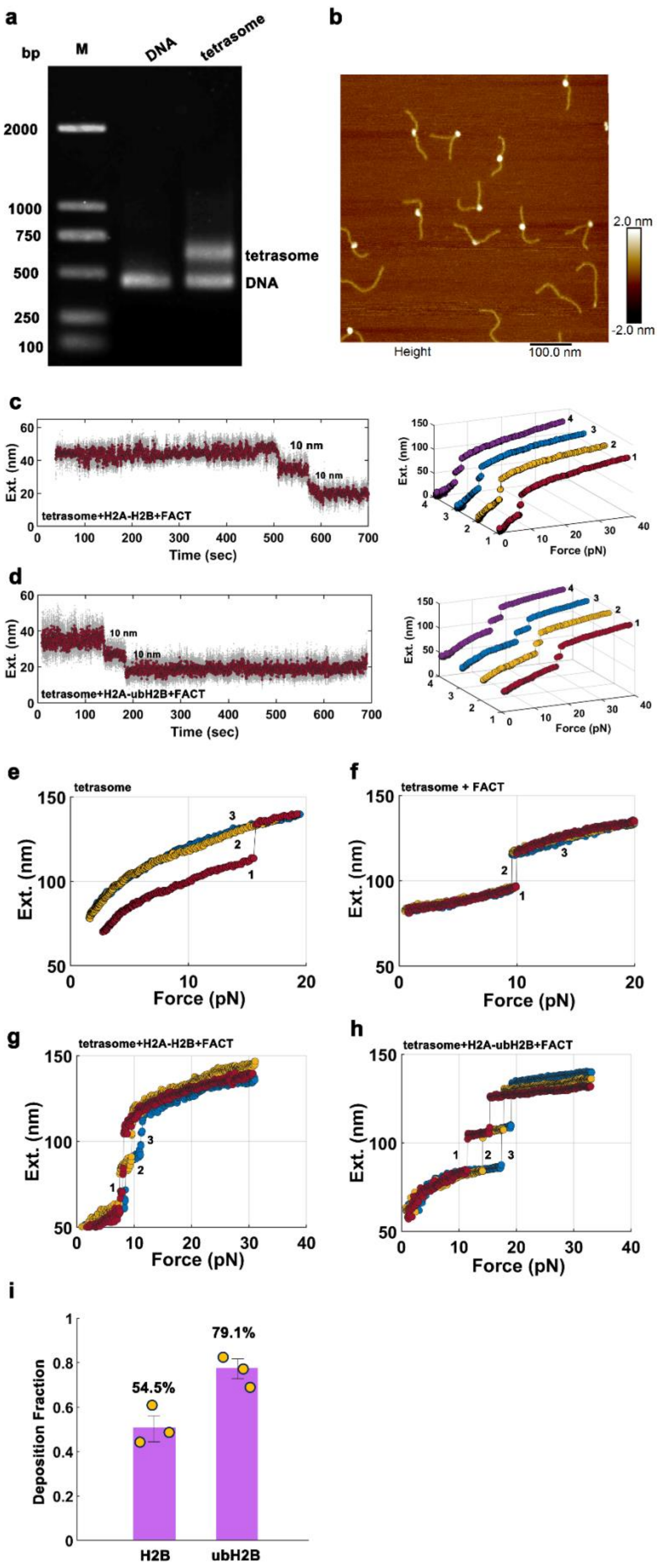

a
bp
M
DNA
tetrasome
2000
1000
750
500
250
100
tetrasome
DNA
b
2.0 nm
-2.0 nm
Height
100.0 nm
c
10 nm
10 nm
tetrasome+H2A-H2B+FACT
Ext. (nm)
Time (sec)
Force (pN)
d
10 nm
10 nm
tetrasome+H2A-ubH2B+FACT
Ext. (nm)
Time (sec)
Force (pN)
e
tetrasome
Ext. (nm)
Force (pN)
f
tetrasome + FACT
Ext. (nm)
Force (pN)
g
tetrasome+H2A-H2B+FACT
Ext. (nm)
Force (pN)
h
tetrasome+H2A-ubH2B+FACT
Ext. (nm)
Force (pN)
i
Deposition Fraction
54.5%
79.1%
H2B
ubH2B

Supplementary Fig. 3: FACT prefers to bind and deposit H2A-ubH2B dimer to form intact nucleosome.

(a) 1% agarose gel electrophoresis analysis of reconstituted tetrasome for magnetic tweezer analysis, with free DNA template (DNA) as control. The data are representative of n=3 biologically independent experiments. The first lane is the marker (M). (b) AFM images of the reconstituted tetrasome. (c) The real-time sequential deposition process of the two H2A-H2B dimers on the reconstituted tetrasome (left), and the repeated stretching measurements of the same molecule after the deposition process (right). The results indicated that FACT deposits two H2A-H2B dimers on the tetrasome to form an intact nucleosome. (d) The real-time sequential deposition process of the two H2A-ubH2B dimers on the reconstituted tetrasome (left), and the repeated stretching measurements of the same molecule after the deposition process (right). The results indicated that FACT deposits two H2A-ubH2B dimers on the tetrasome to form an intact nucleosome. (e-h) The 2D-plots of the repeated stretching measurements of the tetrasome (e), tetrasome with FACT (f), tetrasome with H2A-H2B dimer and FACT (g), and tetrasome with H2A-ubH2B dimer and FACT (h). (i) The quantitative analysis on the deposition ability of FACT for H2A-H2B and H2A-ubH2B dimer onto tetrasome by magnetic tweezers. In the presence of H2A-H2B dimer, 54.5 ±5.3% (mean ± S.D., 150 samples were measured for three biologically independent groups, *n*=3) of the tetrasomes were successfully assembled by FACT to form nucleosome structure. In the presence of H2A-ubH2B dimer, 79.1 ±3.5% (mean ± S.D., 150 samples were measured for three biologically independent groups, *n*=3) of the tetrasomes were assembled to nucleosome. Source data are provided as a Source Data file.

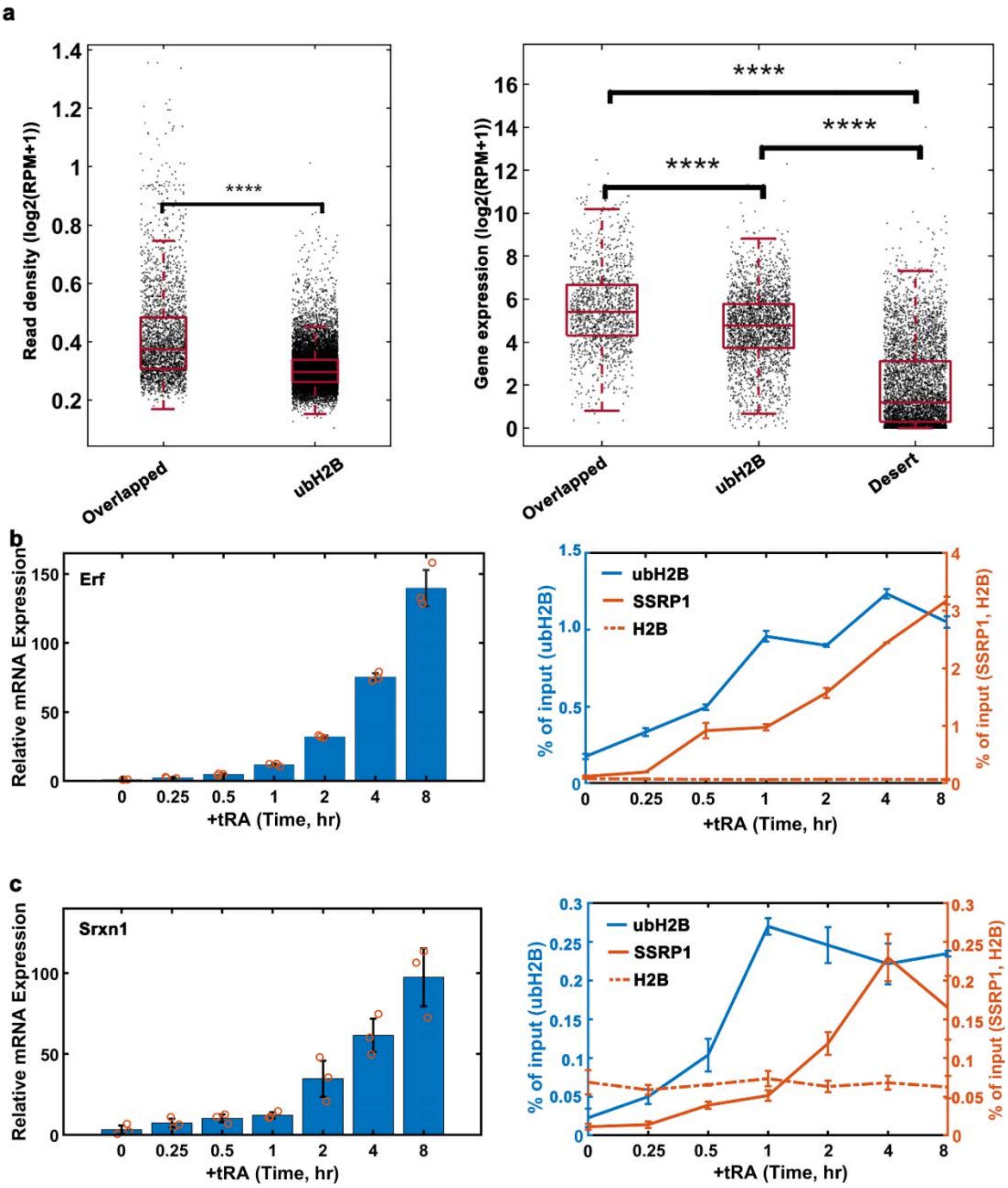

Supplementary Fig. 4: ubH2B recruit FACT to activate gene transcription by genome-wide and specific gene analysis.

(a) Genome-wide analysis on the read density of ubH2B for the genes enriched with ubH2B only and those with SSRP1/ubH2B overlapped (p=0 according to the two-sided student *t* test, *n*=2591 for overlapped and *n*=13672 for ubH2B) (left), and the genome-wide analysis on the expression level of genes enriched with SSRP1/ubH2B overlapped, ubH2B only and ubH2B desert (the genes without ubH2B ChIP-seq peak) (*p*=6.2E-46, 0 and 0 for the comparison of

Overlapped and ubH2B, ubH2B and Desert, Overlapped and Desert, respectively. *n*=1368 for Overlapped, *n*=2461 for ubH2B and *n*=5000 for Desert) (right). ****Significant difference ($p < 0.0001$) according to the two-sided student *t* test. The box plots include the median line (median value indicated), the box denotes the interquartile range (IQR), whiskers denote the rest of the data distribution, and outliers are denoted by points greater than ±1.5×IQR. (b-c) ChIP-qPCR analysis (right) of the level of SSRP1, ubH2B and H2B on the gene body regions of Erf (b) and Srxn1 (c), with the relative levels of mRNA shown (left) at different time points during tRA induction as measured using RT-real time-PCR. The levels were normalized as n-fold changes relative to the values prior to tRA induction. All the data represent means ± S.D. of three independent biological replicates (*n*=3). Source data are provided as a Source Data file.

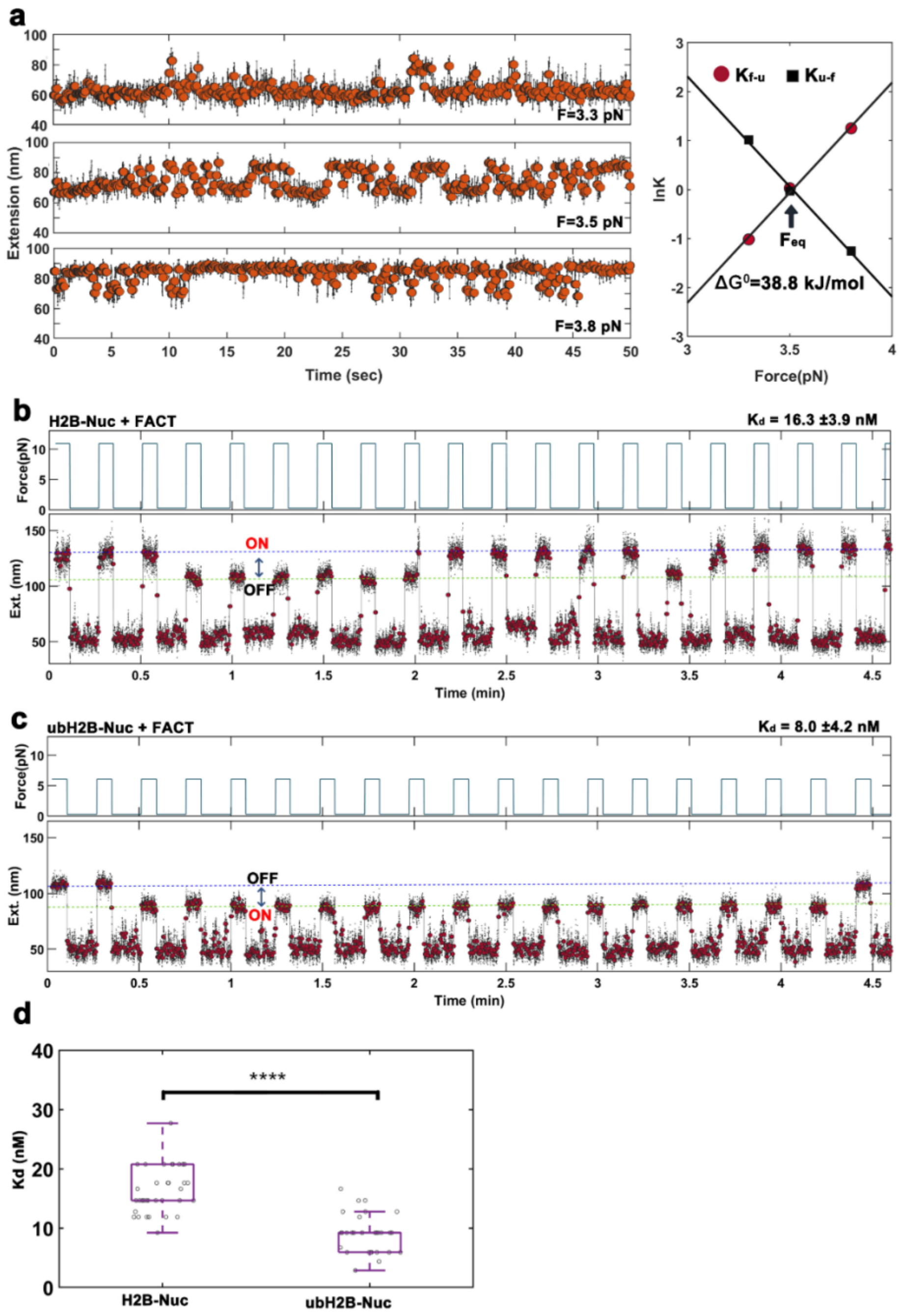

Supplementary Fig. 5: Free energy calculation and *Kd* measurement by single-molecule magnetic tweezers.

(a) Folding/unfolding trajectories of the outer DNA wrap at various tensions (left), and the relationship between the kinetic rate constant and tension (right) for H2B-nucleosome. The free energy cost for unfolding the outer DNA wrap of H2B-nucleosome is calculated to be 38.8 kJ/mol. (b) The typical force-jump measurements on the extension of unmodified H2B-nucleosome at the tension between 0.1 pN and 11 pN reveals the different state of nucleosome.

Nucleosomes bound with FACT (ON) present larger extension than the nucleosomes alone (OFF) at 11 pN due to the disruption of inner DNA wrap. The calculated K_d for FACT binding H2B-nucleosome is 16.3 ± 3.9 nM (mean ± S.D., 1000 stretching cycles for 30 samples). (c) The force-jump measurements on the extension of ubH2B-nucleosome at the tension between 0.1 pN and 6 pN reveals the different state of nucleosome. The ubH2B-nucleosomes alone (OFF) present larger extension than the ubH2B-nucleosomes bound with FACT (ON) at 6 pN due to the disruption of outer DNA wrap. The calculated K_d for FACT binding ubH2B-nucleosome is 8.0± 4.2 nM (mean ± S.D., 980 stretching cycles for 30 samples). (d) The distribution of K_d for FACT's binding to H2B-nucleosome (n=34) or ubH2B-nucleosome (n=30) with significant difference (****Significant difference $p < 0.0001$, p=3.4E-11 according to the two-sided student t test). The box plots include the median line (median value indicated), the box denotes the interquartile range (IQR), whiskers denote the rest of the data distribution, and outliers are denoted by points greater than±1.5×IQR. Source data are provided as a Source Data file.
