# Supplementary material for: H2B ubiquitination recruits FACT to maintain a stable altered nucleosome state for transcriptional activation
Source: Nat Commun. 2023 Feb 10;14:741. doi: 10.1038/s41467-023-36467-3 (PMC9918737; doi:10.1038/s41467-023-36467-3)
Supplement: Supplementary file 3 — Reporting Summary [file 41467_2023_36467_MOESM3_ESM.pdf]

nature portfolio

Corresponding author(s): Wei Li, Ping Chen

Last updated by author(s): Jan 13, 2023

Reporting Summary

Nature Portfolio wishes to improve the reproducibility of the work that we publish. This form provides structure for consistency and transparency in reporting. For further information on Nature Portfolio policies, see our Editorial Policies and the Editorial Policy Checklist.

Statistics

For all statistical analyses, confirm that the following items are present in the figure legend, table legend, main text, or Methods section.

n/a	Confirmed	
☐	☒	The exact sample size (*n*) for each experimental group/condition, given as a discrete number and unit of measurement
☐	☒	A statement on whether measurements were taken from distinct samples or whether the same sample was measured repeatedly
☐	☒	The statistical test(s) used AND whether they are one- or two-sided<br>*Only common tests should be described solely by name; describe more complex techniques in the Methods section.*
☒	☐	A description of all covariates tested
☐	☒	A description of any assumptions or corrections, such as tests of normality and adjustment for multiple comparisons
☐	☒	A full description of the statistical parameters including central tendency (e.g. means) or other basic estimates (e.g. regression coefficient) AND variation (e.g. standard deviation) or associated estimates of uncertainty (e.g. confidence intervals)
☐	☒	For null hypothesis testing, the test statistic (e.g. *F*, *t*, *r*) with confidence intervals, effect sizes, degrees of freedom and *P* value noted<br>*Give P values as exact values whenever suitable.*
☒	☐	For Bayesian analysis, information on the choice of priors and Markov chain Monte Carlo settings
☒	☐	For hierarchical and complex designs, identification of the appropriate level for tests and full reporting of outcomes
☒	☐	Estimates of effect sizes (e.g. Cohen's *d*, Pearson's *r*), indicating how they were calculated

Our web collection on statistics for biologists contains articles on many of the points above.

Software and code

Policy information about availability of computer code

Data collection	AFM images were examined using ScanAsyst Mode of AFM (MultiMode 8 SPM system, BRUKER).<br>LabView software can trace the three-dimensional position of the beads in the flow cell.<br>Real time-PCR and ChIP-qPCR data were collected with StepOnePlusTM realtime PCR system/Thermofisher.<br>ChIP-seq and RNA-seq data that support the findings of this study were downloaded from the Gene Expression Omnibus (GEO: GSE90906, PRJNA604675, GSE153584 and PRJNA643279).
Data analysis	AFM images were processed with Photoshop.<br>The data derived from Labview were analysed by Matlab and plotted with Matlab or GraphPad Prism 6.<br>ChIP-qPCR data were analyzed with Microsoft Excel and plotted with Microsoft Excel or GraphPad Prism 6.<br>ChIP-seq data were mapped to the mouse genome (mm9) using bowtie2 with the default parameters, and the input controls were used in the peak-calling ananlysis for the ChIP-seq data.<br>RNA-seq reads were mapped to the Mus musculus mm9 gene annotation model using hisat2, with the default parameters, and converted to bam files in samtools. Fragments overlapping representative transcripts from annotated genes (genecode vM1) were counted in the featureCounts software. The normalization was performed with R script. Low quality reads and PCR replicates were removed by samtools57 and only uniquely mapped reads which mapping to a unique genomic location and strand were kept. Enriched peaks were called using MACS58. The p-value cutoff for peak detection was 1e-5 with MACS2.<br>Real time-PCR and ChIP-qPCR data were analyzed with Microsoft Excel and plotted with Microsoft Excel or GraphPad Prism 6.

For manuscripts utilizing custom algorithms or software that are central to the research but not yet described in published literature, software must be made available to editors and reviewers. We strongly encourage code deposition in a community repository (e.g. GitHub). See the Nature Portfolio guidelines for submitting code & software for further information.

Data

Policy information about availability of data

All manuscripts must include a data availability statement. This statement should provide the following information, where applicable:

- Accession codes, unique identifiers, or web links for publicly available datasets
- A description of any restrictions on data availability
- For clinical datasets or third party data, please ensure that the statement adheres to our policy

The data were downloaded from Gene Expression Omnibus under the accession ID number GSE90906 (https://www.ncbi.nlm.nih.gov/geo/query/acc.cgi?acc=GSE90906) for the SSRP1 ChIP-seq data, GSE153584 (https://www.ncbi.nlm.nih.gov/geo/query/acc.cgi?acc=GSE153584) for the ubH2B ChIP-seq data, PRJNA604675 (https://www.ncbi.nlm.nih.gov/bioproject/PRJNA604675/) and PRJNA643279 (https://www.ncbi.nlm.nih.gov/bioproject/?term=PRJNA643279) for the RNA-seq expression data. All the data generated in this study are available within the ariticle and its Supplementary Figures with the source data provided as a Source Data file.

Human research participants

Policy information about studies involving human research participants and Sex and Gender in Research.

Reporting on sex and gender	n/a
Population characteristics	n/a
Recruitment	n/a
Ethics oversight	n/a

Note that full information on the approval of the study protocol must also be provided in the manuscript.

Field-specific reporting

Please select the one below that is the best fit for your research. If you are not sure, read the appropriate sections before making your selection.

[x] Life sciences   [ ] Behavioural & social sciences   [ ] Ecological, evolutionary & environmental sciences

For a reference copy of the document with all sections, see nature.com/documents/nr-reporting-summary-flat.pdf

Life sciences study design

All studies must disclose on these points even when the disclosure is negative.

Sample size	No statistical methods were used to predetermine sample sizes. Sample sizes are similar to those used in the filed, as previously reported: Ping et al. Molecular Cell 2018, Wei et al. 2016 Molecular Cell
Data exclusions	no data was excluded from the analyses.
Replication	Each expermient was repeated at least three times in independent experiments. Experimental findings were reproduced reliably.
Randomization	The fields of AFM analysis and single-molecule magnetic tweezers analysis were randomly selected.
Blinding	Blinding was not performed due to the unambiguous nature of measurements and systematic analyses used in these experiments.

Reporting for specific materials, systems and methods

We require information from authors about some types of materials, experimental systems and methods used in many studies. Here, indicate whether each material, system or method listed is relevant to your study. If you are not sure if a list item applies to your research, read the appropriate section before selecting a response.

Materials & experimental systems

n/a	Involved in the study
[ ]	[x] Antibodies
[ ]	[x] Eukaryotic cell lines
[x]	[ ] Palaeontology and archaeology
[x]	[ ] Animals and other organisms
[x]	[ ] Clinical data
[x]	[ ] Dual use research of concern

Methods

n/a	Involved in the study
[x]	[ ] ChIP-seq
[x]	[ ] Flow cytometry
[x]	[ ] MRI-based neuroimaging

Antibodies

Antibodies used

The detail information: Antibodies, company names, catalogue numbers, clone numbers for monoclonal antibodies, antibody dilutions for WBs and ChIP
Anti-H3, Cell signaling, #9715, for WB, 1/5000;
Anti-His, Huaxingbio, HX1804, for WB, 1/2000;
Anti-H2A, Abcam, ab18255, for WB, 1/2000;
Anti-FLAG M2, Sigma, F1804, for WB, 1/2000;
Anti-ubH2B, CST, #5546, for WB,ChIP, 1/2000;
Anti-H2B, Abcam, ab1790, for WB,ChIP, 1/2000;
Anti-SSRP1, BioLegend, 609710, for WB,ChIP, 1/2000;

Validation

All antibodies were commercial in origin and validated by the company. All antibodies used are commonly used in the field and have been validated in previous publications/by the manufacturer.
Anti-H3 (Cell signaling #9715): https://www.cellsignal.com/products/primary-antibodies/histone-h3-antibody/9715
Anti-His (Huaxingbio, HX1804): http://www.huaxingbio.com/pd.jsp?id=340
Anti-H2A (Abcam, ab18255): https://www.abcam.cn/histone-h2a-antibody-chip-grade-ab18255.html
Anti-FLAG M2(Sigma,F1804): https://www.sigmaaldrich.cn/CN/zh/product/sigma/f1804
Anti-ubH2B (CST, #5546): https://www.cellsignal.cn/datasheet.jsp?productId=5546&images=1
Anti-H2B (Abcam, ab1790): https://www.abcam.cn/histone-h2b-antibody-chip-grade-ab1790.html
Anti-SSRP1 (BioLegend, 609710): https://www.labome.com/product/BioLegend/609710.html

Eukaryotic cell lines

Policy information about cell lines and Sex and Gender in Research

Cell line source(s)	R1 cell line (ATCC® SCRC1011™)
Authentication	Identity of R1 cell line was frequently checked by the morphological features, , but not authenticated.
Mycoplasma contamination	Mycoplasma free
Commonly misidentified lines (See ICLAC register)	No commonly misidentified cell lines were used in this study.
